# Vascularization Strategies for Peripheral Nerve Tissue Engineering

**DOI:** 10.1002/ar.23919

**Published:** 2018-10-17

**Authors:** Papon Muangsanit, Rebecca J. Shipley, James B. Phillips

**Affiliations:** ^1^ Biomaterials and Tissue Engineering UCL Eastman Dental Institute, University College London London UK; ^2^ UCL Centre for Nerve Engineering University College London London UK; ^3^ UCL Mechanical Engineering University College London London UK; ^4^ Department of Pharmacology, UCL School of Pharmacy University College London London UK

**Keywords:** peripheral nerve, regeneration, degeneration, therapies, vascularization

## Abstract

Vascularization plays a significant role in treating nerve injury, especially to avoid the central necrosis observed in nerve grafts for large and long nerve defects. It is known that sufficient vascularization can sustain cell survival and maintain cell integration within tissue‐engineered constructs. Several studies have also shown that vascularization affects nerve regeneration. Motivated by these studies, vascularized nerve grafts have been developed using various different techniques, although donor site morbidity and limited nerve supply remain significant drawbacks. Tissue engineering provides an exciting alternative approach to prefabricate vascularized nerve constructs which could overcome the limitations of grafts. In this review article, we focus on the role of vascularization in nerve regeneration, discussing various approaches to generate vascularized nerve constructs and the contribution of tissue engineering and mathematical modeling to aid in developing vascularized engineered nerve constructs, illustrating these aspects with examples from our research experience. Anat Rec, 301:1657–1667, 2018. © 2018 The Authors. *The Anatomical Record* published by Wiley Periodicals, Inc. on behalf of American Association of Anatomists.

## ROLE OF BLOOD VESSELS AND ENDOTHELIAL CELLS IN GUIDING NERVE REGENERATION

**Figure 1:**
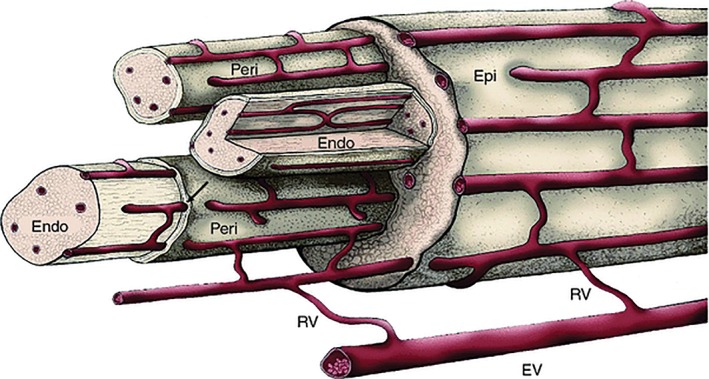
Microcirculation system of a peripheral nerve. The extrinsic vessels (EV) and branch radicular vessels (RV) supply the intrinsic circulation of the vasa nervorum. The intrinsic circulation consists of longitudinally oriented vessels that course to the perineurium (Peri) (Lundborg and Hansson, 1988).

The vasculature plays a fundamental role in supporting the function of peripheral nerves through its supply of blood, oxygen and other nutrients to the cells comprising nerve tissue. Within the body, most cells reside within around 100–200 μm from the nearest vascular source to ensure sufficient delivery of oxygen by diffusion to meet the demands of cellular metabolism (Jain et al., 2005). The vasculature is also crucial in supporting nerve regeneration following injury (Ronald and Robert, 1995). The vascular system of the peripheral nerve, the vasa nervorum, can be categorized into two systems: extrinsic and intrinsic (Best and Mackinnon, 1994). The extrinsic system comprises a series of arteries and veins which run along the surface of a peripheral nerve and mainly supply the epineurial and perineurial regions (Fig. 1). By comparison, the intrinsic system operates independently from the extrinsic system. This system involves small arteries that supply blood and dissolved nutrients to the inner, endoneurial nerve compartment. Several features of the nerve vasculature mean that peripheral nerve tissues are prone to low oxygen conditions. First of all, the inter‐capillary distances in the vasa nervorum are larger than other tissues such as muscle (Low et al., 1989), while glial cell densities are relatively high. This can lead to a reduction in oxygen concentrations compared to other tissues, as a consequence of the balance between longer diffusion distances and significant metabolic demand for oxygen (Bell and Weddell, 1984). Further, the extrinsic vasculature is capable of regulation in response to changes in physiological conditions, (Appenzeller et al., 1984, Zochodne, 2002), whereas vessels of the intrinsic circulation lack the ability to autoregulate (Smith et al., 1977). As a result, if the systemic blood pressure drops, the intrinsic blood vessels fail to compensate for the associated perfusion changes, making the nerve susceptible to ischemia; this can lead to nerve hypoxia and damage.

The fundamental importance of vascularization during nerve regeneration is well‐established. A nerve transection event triggers the break up of the myelin sheath surrounding a nerve fiber, as well as the degeneration of axoplasm distally by the interactions of Schwann cells and macrophages during Wallerian degeneration. Schwann cells distal to the injury then proliferate and form bands of Büngner, which can guide axons growing from the proximal stump toward the distal end. Blood vessels are commonly found to precede Schwann cell migration as well as axonal extension, suggesting the important link between neurite growth and vascular growth (Hobson et al., 1997, 2000).

In a study undertaken in dogs, Tarlove and Epstein (1945) reported that the rate of vascularization seemed to limit the growth of axons into a peripheral nerve graft . In a separate study, cutaneous nerve plexuses of albino rabbits were crushed or transected to analyze the pattern of regeneration, and regions where axonal regeneration were prominent correlated with an abundance of larger blood vessels (Weddell, 1942). Indeed, there is extensive evidence that the architecture and functionality of capillaries in particular facilitate axonal regeneration. Changes in capillary number (Nukada, 1988) and capillary permeability (Weerasuriya, 1988, 1990) associated with successful axonal regeneration suggest that there is an interaction between blood vessels and regenerating axons. For example, the number of endoneurial capillaries increases during regeneration, and the permeability of these capillaries increases, which may encourage clearance of debris and aid elongating axons. Hobson et al. (1997) demonstrated the morphological inter‐relationships between angiogenesis and axonal regeneration in a rat sciatic nerve model (Hobson et al., 1997). The authors reported that Schwann cell migration and axon regeneration were greatest in well‐vascularized regions where vessels were aligned longitudinally, and also that the blood vessel front preceded Schwann cell penetration and axonal regeneration. Vascular endothelial growth factor (VEGF) added in a silicone chamber was shown to significantly increase vascularization and enhance axonal regrowth and Schwann cell proliferation in a rat sciatic nerve injury model, indicating the interdependence between the vascularization and nerve regeneration processes (Hobson et al., 2000).

Endothelial cells, which form the inner lining of blood vessels, are known to synthesize several factors that are supportive of nerve regeneration (Kokaia and Lindvall, 2003, Black et al., 1990). When subventricular zone (SVZ) explants of adult brain are co‐cultured with endothelial cells, neurite outgrowth and migration of neurons are enhanced (Leventhal et al., 1999). Further, vitronectin and heparin sulphate proteoglycans, glycoproteins expressed on the surface of endothelial cells, are involved in neurite activity and growth, as shown using dorsal root ganglion explants in culture (Isahara and Yamamoto, 1995). Human umbilical vein endothelial cells (HUVECs), a widely used source of endothelial cells in angiogenesis studies, have been shown to secrete brain‐derived neurotrophic factor (BDNF), a neurotrophic factor involved in promoting neural cell growth and survival (Nakahashi et al., 2000). Recent studies have shown that molecular cues from nerves influence the branching and network morphology of blood vessels and vice versa, suggesting cross‐talk between neural cells and vascular cells. This is illustrated by the fact that VEGF and Sema3A, which are proteins produced by endothelial cells and neurons respectively, have an opposite effect on neural cells and vascular cells (Bagnard et al., 2001, Miao et al., 1999). Sema3A inhibits both endothelial cell and axonal motility (which is mediated by Neuropilin1‐binding sites), whereas VEGF competes for the same binding sites to stimulate endothelial cell motility. In addition, VEGF antagonizes apoptosis induced by Sema3A. Conditions where neural activity is improved are also seen to trigger angiogenesis. For example, exercise induced increased synaptic activity and a greater density of blood vessels in the cerebellar cortex of adult rats (Black et al., 1990). These studies provide further data to support the relationship between blood vessels and nerves, as well as suggesting that endothelial cells could help promote neurite elongation by providing molecular cues in the early stage of regeneration.

There is also strong evidence that the surfaces of blood vessels directly support Schwann cell migration and hence axon growth (Cattin et al., 2015). This study was performed in a rat sciatic nerve model; when nerves were harvested, blood vessels, Schwann cells and axons were quantified using confocal microscopy. This demonstrated that Schwann cells migrated along the surface of blood vessels and consequently, Schwann cell migration was disrupted when blood vessel network architecture was disorganized.

In summary, the interlinkage between vascularization and nerve regeneration is well established. In the first place, the vasculature provides nutrients such as oxygen for regenerating axons and associated cells, and thus increases long‐term survival. Furthermore, endothelial cells secrete molecules that can be beneficial for neurogenesis and nerve regeneration. Finally, blood vessels also serve as tracks for Schwann cells to migrate along and thus guide axonal growth. As a result, vascularized artificial nerve conduits could be an exciting approach to explore for the repair of peripheral nerve injuries. In the following sections, vascularized nerve constructs are reviewed, then future technological directions including engineered tissues and mathematical modeling are discussed using examples from our current research.

## VASCULARIZED NERVE SUBSTITUTES

Vascular substitutes can be categorized into four main groups: (1) Vascularized nerve grafts, (2) Vascularized grafts by vascular implantation, (3) Blood vessel‐including tubulation, and (4) Biogenic vascularized nerve conduits. Next we discuss these options in turn, including the features that limit their use as clinical repair options. A summary of all approaches is provided in Table 1.

**Table 1:** Summary of the approaches to generate vascularized nerve grafts/conduits and some example studies.

Method	Nerve type and its source of vascularization	Species	Nerve gap	Time point	Outcomes compared with controls	Reference
Vascularized nerve grafts	Vascularized sural nerve graft	Human	50–80 mm	2–41 months	No control Sensitivity was recovered in a satisfactory manner The patients were satisfied with their treatment outcomes	(Rose and Kowalski, 1985)
	Sciatic nerve graft with proximal preserved pedicle	Rabbit	45 mm	5–15 weeks	Conventional nonvascularized sciatic nerve graft as a control Improved remyelination Increased number of axonal fibers	(Restrepo *et al*., 1985)
	Rabbit median nerve graft with brachial vessels	Rabbit	30 mm	10 and 24 weeks	Conventional nonvascularized median nerve graft as a control Greater muscle contraction force and number of axons No statistical difference in CMAP area and muscle weight	(Shibata et al., 1988)
	Rat sciatic nerve with caudal femoral vessels	Rat	15 mm	1–24 weeks	Free sciatic nerve graft as a control Faster motor nerve conduction velocity Greater density of regenerated axons	(Koshima and Harii, 1985)
Vascularized grafts by vascular implantation	Implanting an arteriovenous fistula into the sciatic nerve	Rat	N/A	N/A	N/A	(Cavadas and Vera‐Sempere, 1994)
	Peripheral nerve graft was placed into the groove between the femoral artery and vein for 14 days	Rabbit	N/A	N/A	N/A	(Saray *et al*., 2002)
	PGA nerve conduit vascularized by host superficial inferior epigastric (SIE) for 14 days	Rat	15 mm	1–18 weeks	Nonvascularized conduit graft and an autograft as controls Better functional outcomes andmore myelinated axons than that of the novascularized conduit group Did not achieve the level of reinnervation of autograft	(Iijima et al., 2016)
	Amnion tube placed in contact with the femoral artery and vein for 3 weeks	Rat	10 mm	3 months	Nonvascularized amnion tube as a control Higher number of axons Larger axon diameters Thicker myelin sheath	(Ozcan *et al*., 1993)
Blood vessels‐including tubulation	Insertion of a subcutaneous artery and sciatic nerve in a silicone tube	Rat	5 mm	4, 8, and 15 weeks	Silicone tube only as a control Contain more capillaries than the control More functional and morphological recovery of regenerating nerve	(Kosaka, 1990)
	Silicone tube containing sural vessels	Rat	25 mm	12 and 24 weeks	No control Axons able to regenerate across the gap Reinnervate the tibialis anterior muscle 6 months after operation	(Kakinoki et al., 1997)
	Silicone tube with a subcutaneous artery adjacent to the injured nerve	Human	30–50 mm	6–9 months	No control Out of nine nerves, with a follow‐up of 6–9 months, the results were excellent in five nerves, good in two and poor in two	(Yong‐xiang and Ti‐pei, 1992)
Vascularized biogenic conduits	Silicone rod placed near a sciatic nerve for 8 weeks	Rat	15 mm	8 weeks	Nonvascularized biological conduit as a control Vascularized conduit had significantly improved mean peak amplitudes of the CMAPs No statistical difference between the groups in terms of latencies The myelinated axonal counts was significantly higher	(Yapici et al., 2017)
	*In vivo* formation of biogenic conduit after 4 weeks of implantation parallel to the sciatic nerve	Rat	15 mm	1, 2, 3, and 4 weeks	Autologous nerve graft as a control All groups showed an increase of SFI after 4 weeks with no significant difference Significant higher intraneural amount of fibrous tissue in biogenic conduit Myelin sheaths were thicker	(Penna et al., 2011)
	Silicone rubber rod left *in situ* for at least 3 weeks	Rat	10–12 mm	3 months	No control Good functional recovery of motor fibers	(Lundborg and Hansson, 1980)
	Pseudosheath formed around the silicone tube during the first stage is used as a tunnel to envelope the median nerve graft segment in the second stage	Rat	15 mm	3, 6, and 15 weeks	Conventional median nerve graft as a control Reflex latency was significantly lower than the conventional nerve graft Higher vascularity	(Zadegan et al., 2015)

“N/A” indicates that there is no results of an *in vivo* assessment of the construct.

Abbreviations: CMAP, compound muscle action potential; SFI, sciatic functional index.

## VASCULARIZED NERVE GRAFTS

Vascularized Nerve Grafts (VNGs) involve transplanting nerve grafts complete with vasculature to the site of nerve regeneration and have been developed to address numerous physiological challenges. VNGs could promote intra‐neural perfusion and nutrient delivery in poorly vascularized zones in humans (Schonauer et al., 2012), avoiding the early ischemia of conventional nerve grafts by restoring neural blood vessels.

In a nonvascularized nerve graft (NVNG), neovascularization, the onset of new blood vessel formation at the interface with host tissue, usually occurs by the third day after surgery under supportive conditions (Mani et al., 1992). Conversely, neovascularization in VNGs can occur before the onset of ischemia, due to the presence of vascular structures within the graft (Mani et al., 1992). As a result, VNGs are considered to be superior to NVNGs and have been successfully used in several clinical cases (Shibata et al., 1988, Rose and Kowalski, 1985, Restrepo et al., 1985, Koshima and Harii, 1985). An early example of VNGs can be seen in a study done by St. Clair Strange in 1947 (Strange, 1947). He successfully harvested the ulnar nerve together with its blood supply to graft the median nerve. In 1976, Taylor and Ham introduced a VNG from the superficial radial nerve (based on the radial artery) and used this to repair a median nerve (Taylor and Ham, 1976).

It has been shown that the ability to enhance nerve regeneration of VNGs is superior to NVNGs as length and size of the nerve injury increase, and as vascularization decreases, which can be seen in a scarred wound bed (Koshima and Harii, 1985, Restrepo et al., 1985, Shibata et al., 1988). In a rabbit sciatic nerve model, 45 mm of the nerve was transected and bridged with vascularized sciatic nerve graft with a vascular pedicle and conventional sciatic nerve grafts (NVNGs) (Restrepo et al., 1985). After 8 weeks, vascularized nerve grafts revealed better performances than the conventional graft in terms of number and diameter of nerve fibers. In the functional outcomes aspect, Kanaya et al. (1992) showed in their work that in a rat sciatic nerve model the vascularized sciatic nerve graft group exhibited an improved mean sciatic function index (SFI) compared with the nonvascularized group (Kanaya et al., 1992).

The criteria for selecting which nerves to use as VNGs were primarily based on whether there were dominant arterial pedicles or large supplying vessels that run for a distance outside the nerves. The vascularized radial or ulnar nerve graft has been demonstrated to be successful in several clinical cases by Taylor and Ham, which is ascribed to their dependable blood supply and suitable diameter for microsurgical transfer (El‐Barrany et al., 1999, Townsend and Taylor, 1984). However, due to the fact that a radial nerve's blood supply is the major limb artery (El‐Barrany et al., 1999) and the ulnar nerve is considered important (Townsend and Taylor, 1984), these limit their clinical uses in this context. In general, saphenous and sural nerve grafts are the most popular for grafting because of their dominant arterial pedicles as well as acceptable morbidity at the donor site (El‐Barrany et al., 1999, Staniforth and Fisher, 1978).

Although VNGs have the potential to improve nerve repair, the major limitation is the lack of donor sites. VNGs function well in large nerves, specifically in long gaps. However, to harvest such a large nerve, significant donor‐site morbidity and scarring is inevitably involved. Despite the fact that this hurdle can be solved in part by the use of cable grafting (i.e., several vascularized nerve graft strands), this leads to harvesting more nerve graft strands and thus increased subsequent donor site morbidity (D'Arpa et al., 2015).

## VASCULARIZED GRAFTS BY VASCULAR IMPLANTATION

Several techniques have been studied to achieve the benefits of VNGs but avoid harvesting a nerve graft with its vascular supply to reduce the donor site‐related morbidity described above. One option is to fabricate a nerve graft and preimplant it between a host artery and vein to promote vascularization, then subsequently remove and implant to bridge the repair site (Falco et al., 1992).

Cavadas and Vera‐Sempere examined this approach in 1994 when they attempted to construct a vascularized nerve graft by implanting an arteriovenous fistula into the sciatic nerve in a rat model for 5 weeks (Cavadas and Vera‐Sempere, 1994). Such experimental surgeries have been performed, but their success in terms of promoting neural regeneration needs to be evaluated relative to the VNG approach. Using an arteriovenous bundle, Saray et al. (2002) vascularized sciatic nerve grafts from a rabbits by placing them into the groove between the femoral artery and vein and then removing after 3, 7, or 17 days to analyze. By day 3, the nerve graft was well vascularized and at day 14 the resident Schwann cells and graft integrity were still preserved (Saray et al., 2002). However, this study did not evaluate nerve regeneration using the prefabricated graft. In another study, Ozcan et al. vascularized an amnion tube by placing it between the femoral artery and vein in a rat model for 3 weeks (Ozcan et al., 1993). The microcirculation was successfully visualized at the repair site in all amnion tubes. At 3 months postoperatively, the vascularized amnion conduits showed comparable nerve regeneration to conventional vascularized nerve grafts in terms of number of myelinated axons, axon diameter and myelin thickness. This revealed the ability of vascularized amnion conduits to promote nerve regeneration and bridge the femoral nerve gap. Indeed, the authors concluded that vascularized amnion conduits were superior to their nonvascularized counterparts. More recently, nerve conduits made of polyglycolic acid (PGA) vascularized by host superficial inferior epigastric (SIE) vessels were successfully constructed and this facilitated the regeneration as well as re‐myelination of the rat sciatic nerve (Iijima et al., 2016).

Although prefabricated VNG through AV fistula implantation could be useful clinically for reducing donor‐site morbidity, this approach involves a two‐stage surgery which prolongs the delay before nerve repair and might increase the risk of complications. Therefore, there remain significant opportunities for improving on this approach in developing clinically‐feasible nerve repair solutions.

## BLOOD VESSEL‐INCLUDING TUBULATION

This technique includes native blood vessels directly within a nerve conduit to promote the vascularization process. One of the first studies was conducted by Kakinoki et al. (1997) where a silicone tube containing the sural vessels implanted in a longitudinal orientation was used to bridge a sciatic nerve gap of 25 mm in a rat. The proximal and distal end of the sciatic nerve were sutured to the silicone tube. After 6 months, axons had regenerated across a 25 mm nerve gap in the rats and reinnervated the tibialis anterior muscle (Kakinoki et al., 1997); however, there was no control where tubes without blood vessels were tested, so it is difficult to determine the improvement that resulted from including blood vessels in the tube. In another study, a subcutaneous artery adjacent to the injured nerve was mobilized and then inserted into a silicone tube (Kosaka, 1990). This was used to bridge a 5 mm rat sciatic nerve gap, with the nerve stumps inserted alongside the artery into each end of the tube, and the repair analyzed after 4, 8, and 15 weeks. The results demonstrated more capillaries were present in the vessel‐containing conduits, with rapid capillary‐like structure formation taking place within the tube; by 4 weeks postoperatively, the total number of intraneural microvasculature vessels was almost four times greater than that in a control group. Also, the tube containing the artery exhibited greater morphological and functional recovery of the regenerating nerve.

Although results are promising, studies in this area have tended to use silicone tubes which are non‐biodegradable, may cause compression, negatively affect joint movement and induce synovitis (Lanzetta et al., 1994), so the tubes would need to be removed after several months if this approach were used clinically. To overcome this limitation, biodegradable or biological tube materials are available for clinical use, and it would be interesting to use these in future vascularization studies rather than silicone. Furthermore, their porosity and permeability to oxygen and nutrients may influence the outcomes. In addition, experiments using larger nerve gap lengths and longer follow‐up would be useful to investigate the performance of these approaches effectively.

## VASCULARIZED BIOGENIC CONDUITS

Prefabricated vascularized biogenic nerve conduits can be generated by placing an artificial conduit near a donor nerve or blood supply. After a certain period of time (usually 1–3 weeks), the artificial conduit is enclosed by pseudosheath; in this stage, the tube is removed leaving intact pseudosheath as a vascularized biogenic nerve conduit. This conduit can then be used on its own, filled with biomaterials, or incorporated with a nerve graft.

In many studies, silicone tubes have been used to generate vascularized biogenic conduits. Due to the non‐absorbable characteristics of silicone, a pseudo‐synovial sheath forms around the silicone tube when implanted subcutaneously (Gu et al., 2011), and this was first used as a tendon graft (Culp, 1993). A study in a primate model conducted by Hunter et al. (1983) showed that the pseudo‐synovial sheath consists of three layers: (1) the intima layer which contains cells that provide a soft and sliding surface, (2) the media layer that was dense with collagen and vascularity, and (3) the adventitia layer which was composed of vascular fibrous tissue. This pseudo‐synovial sheath provides a highly vascular structure throughout all layers (Hunter et al., 1983). There is an evidence suggesting that this vascularized pseudo‐synovial sheath could be used as a nerve graft (Wolford and Stevao, 2003). Lundborg and Hansson used this pseudo‐synovial sheath as a biogenic nerve conduit to repair peripheral nerve injuries with gaps of 10 to 12 mm in rats (Lundborg and Hansson, 1980). Subsequently, several studies have reported success with biogenic conduits to repair nerve injuries in rat models (Penna et al., 2011, Yapici et al., 2017, Zadegan et al., 2015).

In one study, a polyvinyl chloride (PVC) tube was implanted parallel to the rat sciatic nerve (Penna et al., 2011). After 4 weeks, this PVC tube had been covered with pseudo‐synovial sheath and had a higher number of blood vessels per cross section compared to an autologous nerve graft. When used to repair a 15 mm nerve gap injury, there was successful regeneration with significantly higher axon area than an autologous nerve graft. Recently, Yapici et al. (2017) fabricated vascularized biological conduits by placing a silicon rod next to the sciatic nerve of the rat (Yapici et al., 2017). Without damaging the fibrovascular sheath formed around the rod, the silicone rod was removed at the 8‐week time point leaving the vascular sheath which was then used as a nerve conduit. This provided better nerve regeneration when compared to non‐vascularized conduits (connective tissue sheath from a silicone tube placed to the dorsum of the rat) and autografts, both histologically and electrophysiologically. This vascular sheath was also shown to reduce adhesion and scar formation compared to non‐vascularized conduits.

Vascularized biogenic conduits avoid sacrificing a donor nerve with its blood vessel supply (nerve tissue transfer), as well as decreasing the adverse effects of scarring at the nerve injury site. Furthermore, a biogenic conduit provides the soft lumen, good vascularization, and a stable structure that could be effectively used to envelop the nerve graft segment (Zadegan et al., 2015). However, these conduits require a two‐stage procedure for implementation clinically, and the delay associated with the prevascularization step would inevitably limit nerve regeneration capacity in a clinical setting.

Overall, vascularized nerve substitutes are generally accepted as a valuable reconstructive tool for nerve repair. However, the donor site morbidity associated with the vascular substitutes presents a significant limitation to clinical uptake. Therefore, several techniques have been developed to try to reduce this donor site‐related morbidity while enhancing nerve regeneration compared to the gold‐standard, as described in the four sections above. Despite better outcomes coming from those substitutes, problems associated with the long delay required to generate prevascularized nerve conduits, and the limited anatomical supply, still remain.

## ENGINEERED NEURAL TISSUES

Tissue engineering using vascular endothelial cells and biomaterial scaffolds to prefabricate a vascularized nerve construct provides an alternative approach to vascularization in long gap nerve repair. There are not many studies that report specifically the engineering of vascularized nerve tissue constructs. One study demonstrated that by co‐culturing Schwann cells and vascular endothelial cells within fiber‐reinforced 3D composite scaffolds, vascularized nerve engineered constructs can be formed (Gao et al., 2013). According to that study, the combination of Schwann cells and vascular endothelial cells, both taken from rabbits and mixed at a ratio of 2:1, were cultured and then this mixed cell population was injected at both ends of the fiber‐reinforced scaffolds to form a vascularized tissue engineered nerve construct. These constructs were assessed in sciatic nerve injury rabbit models of 20 mm gap at three different postoperative periods (4, 8, and 16 weeks) and were beneficial in promoting nerve repair in terms of conduction velocity, number of nerve fibers and myelin thickness. In another study conducted by Gingras et al. (2003), a tissue‐engineered model of peripheral nerve regeneration was developed which consisted of collagen‐chitosan sponges populated with human endothelial cells and/or fibroblasts (Gingras et al., 2003). When the endothelial cells were present, there was a significant increase in neurite elongation after 14 days in culture, supporting the utility of having endothelial cells in an engineered nerve construct.

**Figure 2:**
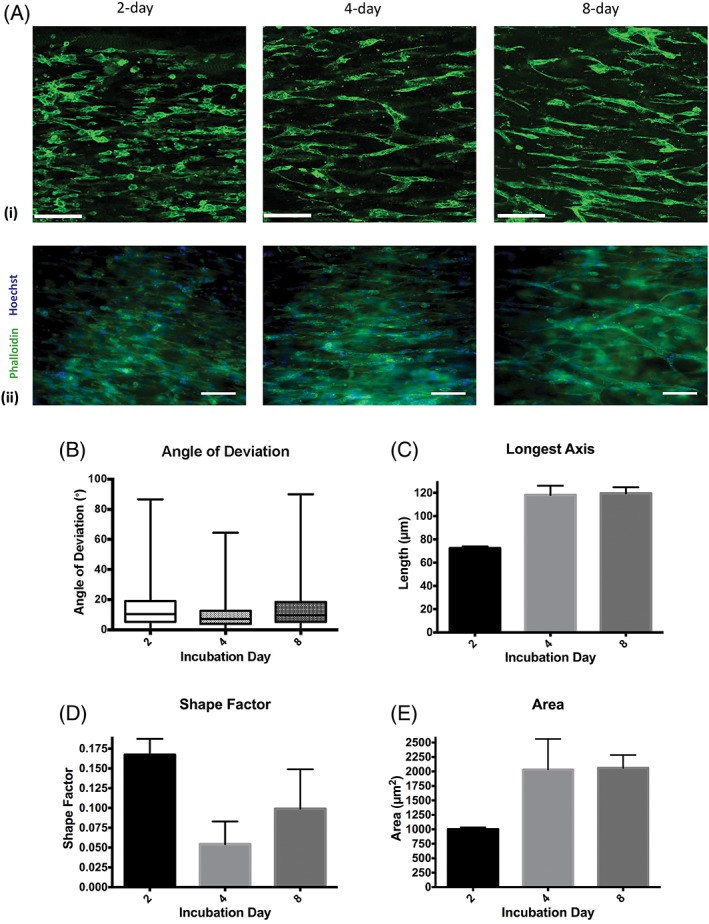
Self‐alignment of HUVECs and formation of tube‐like structures within tethered collagen gels. Confocal micrographs (**A(i)**) and immunofluorescence images (**A(ii)**) show aligned HUVECs forming vascular networks after 2, 4, and 8 days in culture, z‐distance 20 μm, step size 1 μm. Three‐dimensional image analysis was used to calculate the angle of deviation between HUVEC/tube alignment and the longitudinal axis of the gel **(B)**. Boxes show interquartile range and median values, whiskers indicate maximum and minimum angles (N = 3 gels). The length of tube‐like structures **(C)**, shape factor which determine how round the object is (values closer to 1 indicate more rounded shape) **(D)** and surface area **(E)** were compared in 2‐day, 4‐day, and 8‐day cultured gels. Graphs show mean value ± SEM. (N = 3 gels). Scale bars in (A(i)) = 120 μm and in (A(ii)) = 100 μm.

Combining the concept that vascularization of nerve grafts can improve their effectiveness with the observations that endothelial cell‐seeded constructs are beneficial and that vascular structures can guide Schwann cell migration, leads to the hypothesis that engineering vascular structures within scaffolds could help to improve nerve repair construct design. Several approaches have been studied to generate microvascular networks *in vitro*. Many studies attempted to form patterns with cells using techniques such as soft lithography (Whitesides et al., 2001), micropatterning (Folch and Toner, 2000), and photolithography (Kaihara et al., 2000). Another approach involves using the ability of endothelial cells to form networks within scaffolds. Endothelial cells can spontaneously form capillary‐like networks when cultured in 3D under permissive conditions *in vitro*. They have been demonstrated to be able to form tube‐like structures within hydrogels either by co‐culturing with other cell types like fibroblasts or pericytes, or by adding angiogenic growth factors (Berthod et al., 2006, Berthod et al., 2012). In our lab, we successfully created aligned tube‐like vascular structures within collagen hydrogels using human umbilical vein endothelial cells (HUVECs) (Fig. 2). The engineered vascular tissue was constructed using the same approach as previously applied to create aligned cellular collagen gels containing Schwann cells or other therapeutic cells for nerve repair termed Engineered Neural Tissue (EngNT) (Georgiou et al., 2013, Georgiou et al., 2015, Martens et al., 2014, O'Rourke et al., 2015, Sanen et al., 2017). In this process, cells embedded within tethered collagen hydrogels self‐align in response to tension generated through natural cell‐matrix interactions, then the aligned cellular gels are stabilized using plastic compression (Brown et al., 2005).

**Figure 3:**
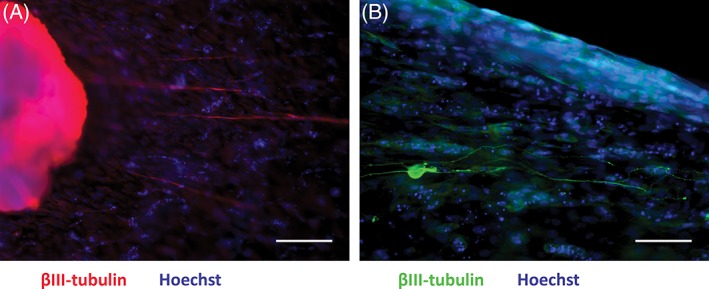
Aligned endothelial cells within collagen gels support and guide neurite growth *in vitro*. Neurites from explanted rat DRG **(A)** and from dissociated DRG neurons **(B)** elongated along the longitudinal axis of the gel and were associated with the aligned endothelial cells. Scale bars in A, B = 100 μm.

These collagen gels containing aligned tube‐like structures have been shown to promote neurite outgrowth *in vitro* when co‐cultured with dorsal root ganglion (DRG) explants (Fig. 3A) and dissociated DRGs (Fig. 3B), suggesting their ability to support and guide neuronal regeneration.

Besides using endothelial cells to generate vascular structures within tissue‐engineered nerve constructs, vascularization of engineered constructs can be promoted through embedding angiogenic factors. This can enhance local angiogenesis and promote integration of constructs with the host vasculature. In nerve tissue engineering studies, vascular endothelial growth factor (VEGF) has been added to nerve constructs and used to repair rat sciatic nerves, resulting in increased numbers of blood vessels as well as axons when compared to constructs without added VEGF (Hobson, 2002, Hobson et al., 2000, Mohammadi et al., 2013, Sondell et al., 1999). Furthermore, there are several growth factors used to induce and support vascularization for tissue engineering that can be applied for nerve constructs such as basic fibroblast growth factor (bFGF) and platelet‐derived growth factor (PDGF) (Carmeliet, 2000, Nomi et al., 2002). Novel angiogenic small molecules have also been investigated, such as in a study by Wieghaus et al. (2006) which developed SC‐3‐149, a non‐peptide‐based inducer, that can increase the proliferation of human microvascular endothelial cells and network formation *in vitro* (Wieghaus et al., 2006). Another approach is to genetically manipulate cells to overexpress a proangiogenic factor, the most widely used of which is VEGF. Geiger et al. (2007) used bone marrow stromal cells (BMSCs) transfected with the VEGF plasmid to enhance vascularization of a bone substitute. Their results showed that genetic modification of BMSCs significantly improved vascularization and osteogenesis for *in vivo* bone healing (Geiger et al., 2007).

## MATHEMATICAL MODELING AS A TOOL TO AID IN THE FABRICATION OF VASCULARIZED NERVE CONSTRUCTS

Vascularized nerve constructs have potential to contribute to the next generation of living replacement tissues; however, there are numerous outstanding questions related to their optimal design and fabrication. For a vascular nerve repair construct to be successful, it must support angiogenesis *in vivo* while minimizing loss of valuable therapeutic cells, and also provide the physical and chemical cues to support axon regeneration. This requires sensitive consideration of the spatial and temporal distribution of material, cells, and chemical factors which is challenging to do in conventional experimental settings in isolation. For example, cell populations such as endothelial or therapeutic Schwann cells are usually added to constructs at standard densities, without exploring the effect of varying these seeding densities on regeneration outcomes. Similarly, the spatial distribution of seeded cells is rarely considered; typically cells are cultured on the luminal surface of a construct tube, distributed throughout the lumen of the tube in suspension, or grown on/in materials packed inside the construct. The seeded cell density and its spatial distribution is critically important, as they determine how gradients of oxygenation and vascular growth factors are established over time *in vivo*. In turn, these gradients regulate hypoxia, cell death rates, and the success of angiogenesis in vascularizing the construct.

These features are highly challenging to characterize experimentally due to the cost and time limitations of extensive *in vitro* and *in vivo* experimentation. However, combining mathematical modeling with the experimental programme has significant potential to streamline the design process and accelerate the pipeline toward clinical translation, and this is the approach taken in our lab (Coy et al., 2016). Such mathematical models should be developed in parallel with preliminary experimentation, so that iteration between model predictions and experimental measurements can inform parameters (e.g., cell proliferation or oxygen consumption rates, etc.) within the mathematical framework, and outputs of the mathematical models can inform the parameter range to explore experimentally. This parameterized framework can then be used to run virtual (or *in silico*) tests of design features (such as seeded cell densities and their spatial distribution), before prioritizing the most promising designs for more extensive experimental testing. In this way, the programme is focused on the experiments that will generate the most meaningful data and promising outcomes.

An experimental‐computational approach has the potential to capitalize on the diverse and advanced tissue engineering, biomaterial and cell technologies now available, and streamline the process of combining them to maximize regeneration using vascularized nerve constructs. It would also exploit a growing literature in computational modeling of blood flow, tissue oxygenation, and angiogenesis (Anderson and Chaplain, 1998, Secomb et al., 2013) for the benefit of peripheral nerve tissue engineering.

## CONCLUSIONS

Vascularization is likely to be an important component in the development of successful new nerve repair strategies. It is required in order for the living components of cellular constructs to survive following implantation, as well as being involved directly in supporting and guiding Schwann cell migration and neuronal regeneration (Iijima et al., 2016, Auger et al., 2013, Cattin et al., 2015). Various techniques have been investigated including free and pedicled vascularized nerve grafts, vascular implantation, blood vessel‐including tubulation, and vascularized biogenic conduits. These in turn inform the development of vascularized tissue‐engineered nerve constructs, providing new opportunities to develop sophisticated living artificial tissue.
